# Integrated [^18^F]FDG PET/MRI demonstrates the iron-related bone-marrow physiology

**DOI:** 10.1038/s41598-020-70854-w

**Published:** 2020-08-17

**Authors:** Tetsuya Tsujikawa, Hiroshi Oikawa, Toshiki Tasaki, Naoko Hosono, Hideaki Tsuyoshi, Mahmudur G. M. Rahman, Yoshio Yoshida, Takahiro Yamauchi, Hirohiko Kimura, Hidehiko Okazawa

**Affiliations:** 1 Biomedical Imaging Research Center, University of Fukui, 23-3 Matsuoka-Shimoaizuki, Eiheiji-cho, Fukui 910-1193 Japan; 2 Department of Hematology and Oncology, Faculty of Medical Sciences, University of Fukui, Fukui, Japan; 3 Department of Obstetrics and Gynecology, Faculty of Medical Sciences, University of Fukui, Fukui, Japan; 4 Department of Radiology, Faculty of Medical Sciences, University of Fukui, Fukui, Japan; 5 Department of Biomedical Engineering, Khulna University of Engineering and Technology, Khulna, Bangladesh

**Keywords:** Bone marrow cells, Predictive markers, Bone

## Abstract

We identified predictors for bone-marrow [^18^F]FDG uptake and MR signals among complete blood count, C-reactive protein (CRP), and anthropometric factors, and demonstrated the bone-marrow physiology using integrated [^18^F]FDG-PET/MRI. 174 oncology patients without bone-marrow lesions underwent whole-body [^18^F]FDG-PET/MRI. The standardized uptake value (SUV), apparent diffusion coefficient (ADC), proton density fat-fraction (PDFF), and a reciprocal of T2* relaxation time (R2*) were measured in lumbar vertebrae (L3–5) and bilateral ilia. Vertebrae, pelvis, and ribs were evaluated by 3-point visual scoring on DWI. The association of the PET/MR features with the predictors was examined. Multi-regression analyses identified CRP as the strongest predictor for lumbar and iliac SUVs (standardized coefficient: β = 0.31 and β = 0.38, respectively), and for lumbar and iliac R2* (β = 0.31 and β = 0.46, respectively). In contrast, age was the strongest factor influencing lumbar and iliac ADCs (β = 0.23 and β = 0.21, respectively), and lumbar and iliac PDFFs (β = 0.53 and β = 0.54, respectively). Regarding DWI-visual scores, age was the strongest predictor for vertebrae (β = − 0.47), and the red cell distribution width (RDW) was the strongest predictor for pelvis and ribs (β = 0.33 and β = 0.47, respectively). The bone-marrow [^18^F]FDG uptake and R2* reflect anemia of inflammation (increased granulopoiesis and reduced iron metabolism), whereas bone-marrow DWI and PDFF reflect age and anemia-responsive erythropoiesis.

## Introduction

Positron emission tomography with 2-[^18^F]-fluoro-2-deoxy-*D*-glucose ([^18^F]FDG PET) and functional magnetic resonance imaging (MRI) such as diffusion-weighted imaging (DWI), proton density fat fraction (PDFF) and a reciprocal of the T2* relaxation time (R2*) maps have been separately used for evaluating bone-marrow abnormality in patients with malignant lymphoma, multiple myeloma, and other hematological disorders^1–4^.[^18^F]FDG PET reveals glycolytic activity, DWI reflects the restricted random movement of extra-cellular water protons (cellular density), PDFF measurements assess the fat content, and R2* reflects the level of iron deposition. The recent introduction of integrated PET/MRI has enabled simultaneous whole-body bone-marrow PET and functional MR imaging in bone-marrow disorders^5,6^. Therefore, physiological factors influencing bone-marrow [^18^F]FDG uptake and MR signals and the associations should be clarified for accurate diagnosis.

We recently reported that age, hemoglobin (Hb), and the red cell distribution width (RDW) are the predominant predictors of bone-marrow signals on DWI^7^. A lower ADC and higher visibility of bone-marrow DWI were associated with a younger age, anemia, and increased erythropoietic activity (higher RDW). On the other hand, bone-marrow [^18^F]FDG uptake was reported to be associated with age, white blood cell (WBC), serum C-reactive protein (CRP), and treatment with granulocyte colony-stimulating factor (GCSF) or erythropoietin^8–13^. However, no previous study has simultaneously evaluated and compared the [^18^F]FDG uptake, DWI signals, PDFF, and R2* of bone marrow in the same subjects.

The objectives of this study were: first, to identify the factors influencing bone-marrow [^18^F]FDG uptake and MR signals among anthropometric factors, complete blood count (CBC), and CRP in patients; and second, to demonstrate the bone-marrow physiology using integrated [^18^F]FDG PET/MRI.

## Patients and methods

### Patient population

We retrospectively reviewed the medical records of all tumor patients who underwent whole-body PET/MRI with [^18^F]FDG in our institute between February 2017 and August 2018. Patients were eligible for the study if they fulfilled the following criteria: (1) whole-body DWI, ADC, PDFF, and R2* maps were obtained, (2) CBC and CRP data measured within one week of the scan were available, and (3) normal renal function was confirmed^7^. Normal renal function is defined as an estimated glomerular filtration rate of (eGFR) > 50 mL/min/1.73 m^2^; this condition is necessary because renal insufficiency is associated with impaired erythropoietic response to anemia. Patients were excluded from this study if they had a hematological disorder (myeloma, leukemia, myelodysplastic syndrome, lymphoma, etc.), or history of chemotherapy, radiotherapy, blood transfusion, or use of GCSF.

One hundred and seventy-four patients (155 females, 19 males; mean age = 56.6 ± 15.4 years) were identified. The details were 151 patients with gynecological tumor (mean age = 55.4 ± 15.7 years), 13 with rectal cancer (2 females, 11 males; mean age = 64.3 ± 9.0 years), and 13 with head and neck cancer (2 females, 8 males; mean age = 64.2 ± 13.9 years).

### Whole-body PET/MRI

#### PET scan and Dixon-based MR-AC

Patients fasted for at least 4 h prior to an intravenous injection of 200-MBq [^18^F]FDG. Fifty minutes after the injection, patients were transferred to an integrated 3.0-T PET/MR scanner (Signa PET/MR, GE Healthcare, Waukesha, WI, USA)^7^. Anatomic coverage was from the vertex to mid-thigh. PET acquisition was performed in the 3D mode with 5.5 min/bed position (89 slices/bed) in 5–6 beds with a 24-slice overlap. The duration of 5.5 min/bed position was selected to accommodate the MRI sequences acquired at each bed. A 2-point Dixon 3D volumetric interpolated T1-weighted fast spoiled gradient echo sequence (TR/TE1/TE2: 4.0/1.1/2.2 ms; FOV 50 × 37.5 cm; matrix 256 × 128; slice thickness/overlap: 5.2/2.6 mm; 120 image/slab; imaging time: 18 s) was acquired at each table position and used to generate MR attenuation correction (MR-AC) maps. Dixon-based MR-AC recognizes body tissues as soft tissue, fat, or air. PET data were reconstructed with ordered subset expectation maximization (OSEM) selecting 14 subsets and 3 iterations, and post-smoothing with a 3-mm Gaussian filter. Reconstructed images were then converted to semiquantitative images corrected by the injected dose and subject’s body weight (= standardized uptake value: SUV).

#### MR sequence parameters

Additional MR sequences were acquired in the axial plane^7^. DWI was performed using a single-shot echoplanar imaging (EPI) sequence under free breathing (TR/TE: 5,000/61 ms; b values: 0, 800 s/mm^2^; FOV 576 × 345 mm; matrix 128 × 128; slice thickness/overlap: 6/0 mm; 40 image/bed; imaging time: 2 min 30 s). A short inversion time inversion recovery (STIR) pre-pulse was used for fat suppression on whole-body DWI because of its insensitivity to magnetic field inhomogeneity^14^, and a selective water excitation (spectral-spatial radiofrequency, SSRF) pre-pulse was used together with STIR to yield a higher signal-to-noise ratio (SNR) in the abdomen and pelvis^15^. PDFF and R2* measurements were performed using an iterative decomposition of water and fat with echo asymmetry, and a least-squares estimation quantitation sequence (IDEAL-IQ) (a quantitative chemical shift-based water-fat separation method with a multiecho gradient echo^16^; TR/TEs: 7.1/0.9–5.3 ms, 6 echoes; FOV 500 × 300 mm; matrix 256 × 192; slice thickness/overlap: 6/0 mm; 34 image/bed; imaging time: 20 s). ADC, PDFF, and R2* maps were generated, and used in subsequent assessments.

### Quantitative image assessment

**Figure 1:**
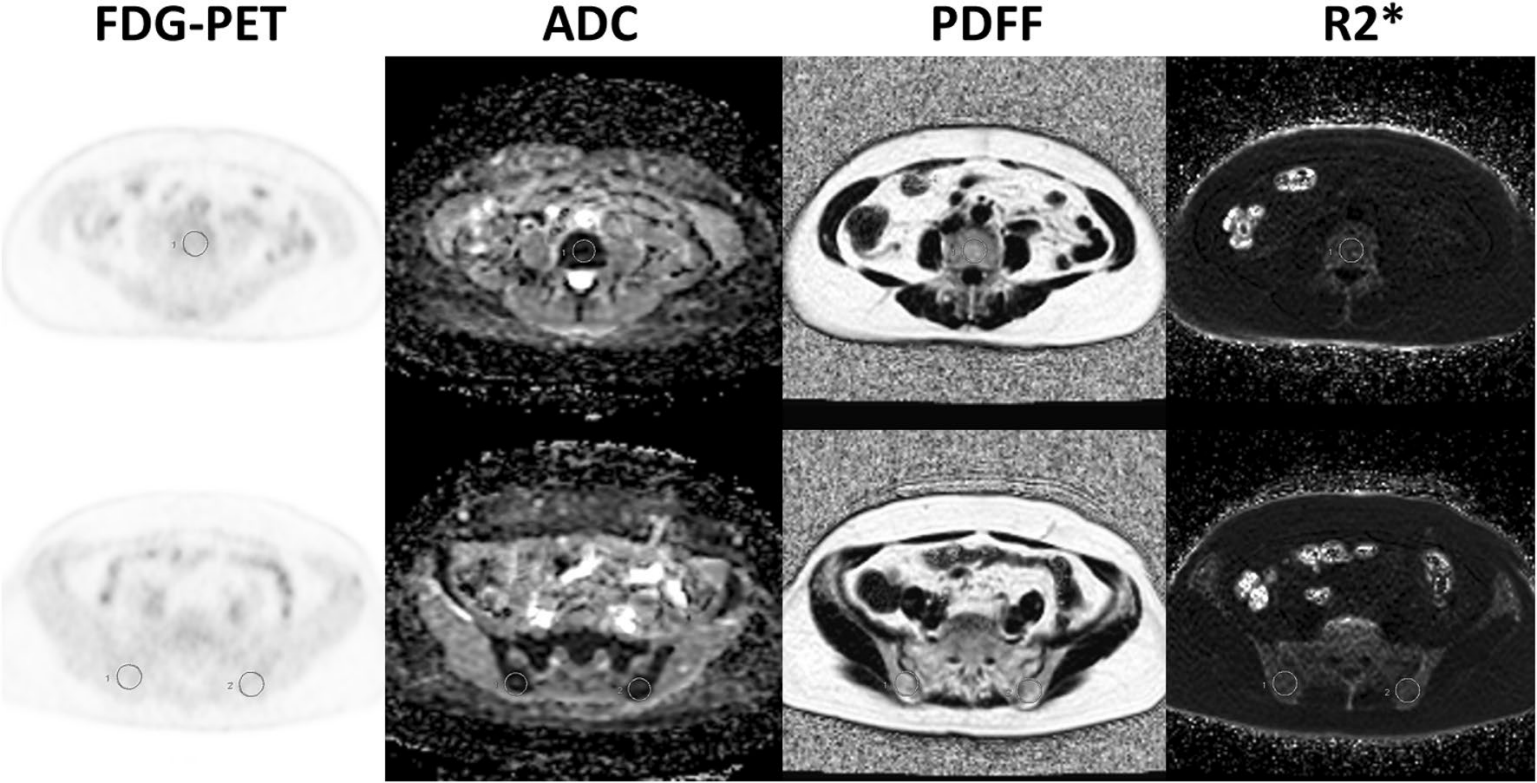
ROIs placement on L4 (top) and bilateral ilia (bottom).

PET and MR images were transferred to the GE workstation (AW 4.6) and evaluated with matched spatial registration. Circular regions of interest (ROIs) with a fixed diameter of 20 mm were placed on lumbar vertebrae (L3–5) and bilateral posterior iliac crest^7^ (Fig. 1). The patients had no bone metastases in the areas where ROIs were drawn.[^18^F]FDG-SUVmean, ADC, PDFF, and R2* were measured, and averaged in L3–5 and iliac bones by the agreement of an experienced radiologist and hematologist (TeT and ToT with 17 and 15 years of experience, respectively). In addition, ribs, vertebrae, and pelvic bones were evaluated by 3-point visual scoring on maximum intensity projection (MIP) DW images with b = 800. A score of 1 was assigned to images in which bones were invisible, score of 2 to images in which bones were partially visible, and score of 3 to images in which bones were fully visible. The associations of [^18^F]FDG-SUV, ADC, DWI-visual scores, PDFF, and R2* with anthropometric and blood-related data were examined. Anthropometric data included age, height, and body weight, except that body weight was excluded in the evaluation of [^18^F]FDG-SUV because it is a factor related to SUV calculation. Gender was excluded as a factor due to the predominance of female patients in this study. Blood-related data evaluated in this study, with the abbreviation and normal range in parentheses, are as follows: white blood cell (WBC, 3.3–8.6 × 10^3^/μL), hemoglobin (Hb, 13.7–16.8 g/dL), red cell distribution width (RDW, 11.0–15.0%), platelet count (Plt, 158–348 × 10^3^ /μL), platelet distribution width (PDW, 15.0–17.0%), and serum C-reactive protein (CRP, 0–0.14 mg/dL).

Stepwise multiple regression analysis was performed to find the most predominant predictive factors for [^18^F]FDG-SUV, ADC, DWI-visual scores, PDFF, and R2* from anthropometric and blood-related data. Regression analyses between the top predictors and [^18^F]FDG-SUV, ADC, PDFF, and R2* were performed using Pearson’s correlation coefficient, and between the top predictors and DWI-visual scores using Spearman’s correlation coefficient. All statistical analyses were performed using SPSS statistics version 22. *p* < 0.05 was considered to be significant.

### Ethical approval

All procedures performed in studies involving human participants were in accordance with the ethical standards of the institutional and/or national research committee and with the 1964 Helsinki declaration and its later amendments or comparable ethical standards. This retrospective study was approved by the ethics committee of the Faculty of Medical Sciences, University of Fukui (No. 20170206), and the requirement to obtain formal informed consent was waived.

## Results

### Predictor of bone-marrow [^18^F]FDG-SUV and MR signals

Bone-marrow [^18^F]FDG-SUV, ADC, PDFF, and R2* for each region are presented in Table 1. The results of multiple regression analyses to identify the strongest predictors for bone-marrow [^18^F]FDG-SUV, ADC, DWI-visual scores, PDFF, and R2* are shown in Table 2. CRP was the strongest factor influencing lumbar and iliac SUVs (standardized coefficient: β = 0.31, Pearson’s correlation coefficient: r = 0.37, and β = 0.38, r = 0.46, respectively), and lumbar and iliac R2* (β = 0.31, r = 0.31, and β = 0.46, r = 0.47, respectively). In contrast, age was the strongest factor influencing lumbar and iliac ADCs (β = 0.23, r = 0.25, and β = 0.21, r = 0.26, respectively), and lumbar and iliac PDFFs (β = 0.53, r = 0.55, and β = 0.54, r = 0.56, respectively). Regarding DWI-visual scores, age was the strongest predictor for vertebrae (β = − 0.47, Spearman’s correlation coefficient: r = − 0.41), and RDW was the strongest predictor for the pelvis and ribs (β = 0.33, r = 0.41, and β = 0.47, r = 0.47, respectively).

**Table 1:** Regional [^18^F]FDG-SUV ADC, PDFF, and R2*.

Region	[^18^F]FDG-SUV (g/mL)	ADC (× 10^–6^ mm^2^/s)	PDFF (%)	R2* (Hz)
Third lumbar vertebra (L3)	1.45 ± 0.51 (0.54–3.63)	478 ± 128 (226–926)	45.6 ± 14.3 (13.9–78.1)	126.5 ± 40.1 (14.4–307.0)
Fourth lumbar vertebra (L4)	1.36 ± 0.48 (0.38–3.51)	465 ± 116 (225–806)	46.4 ± 15.3 (15.4–97.4)	125.1 ± 36.1 (56.5–240.5)
Fifth lumbar vertebra (L5)	1.32 ± 0.47 (0.40–3.40)	471 ± 139 (230–966)	47.2 ± 15.3 (13.2–81.4)	128.7 ± 34.2 (57.5–272.4)
Average (L3–5)	1.38 ± 0.48 (0.56–3.51)	472 ± 115 (228–789)	46.4 ± 14.5 (15.1–80.5)	126.8 ± 34.2 (60.8–263.4)
Right posterior iliac crest	1.00 ± 0.46 (0.23–3.33)	463 ± 131 (162–878)	61.2 ± 16.4 (21.5–93.0)	116.0 ± 27.2 (72.4–263.5)
Left posterior iliac crest	0.97 ± 0.43 (0.23–2.92)	475 ± 134 (214–891)	61.3 ± 16.4 (22.8–92.2)	118.4 ± 26.9 (70.4–269.2)
Average (bilateral)	0.98 ± 0.44 (0.23–3.13)	469 ± 125 (210–851)	61.3 ± 16.1 (22.2–91.5)	117.2 ± 26.2 (73.3–266.4)

Values are the mean ± SD with ranges in parentheses.

**Table 2:** Top predictors of bone marrow [^18^F]FDG-SUV and MR signals.

Parameters	Predictor	β	r
SUV (L3–5)	CRP	0.31 (*p* < 0.0005)	0.37 (*p* < 0.0001)
SUV (ilium)	CRP	0.38 (*p* < 0.0001)	0.46 (*p* < 0.0001)
ADC (L3–5)	Age	0.23 (*p* < 0.01)	0.25 (*p* < 0.005)
ADC (ilium)	Age	0.21 (*p* < 0.05)	0.26 (*p* < 0.001)
DWI-VS (vertebrae)	Age	− 0.47 (*p* < 0.0001)	− 0.41 (*p* < 0.0001)
DWI-VS (pelvis)	RDW	0.33 (*p* < 0.0005)	0.41 (*p* < 0.0001)
DWI-VS (rib)	RDW	0.47 (*p* < 0.0001)	0.47 (*p* < 0.0001)
PDFF (L3–5)	Age	0.53 (*p* < 0.0001)	0.55 (*p* < 0.0001)
PDFF (ilium)	Age	0.54 (*p* < 0.0001)	0.56 (*p* < 0.0001)
R2* (L3–5)	CRP	0.31 (*p* < 0.005)	0.31 (*p* < 0.005)
R2* (ilium)	CRP	0.46 (*p* < 0.0001)	0.47 (*p* < 0.0001)

DWI-VS: DWI-visual score.

β: standardized coefficient calculated by a stepwise regression.

r: Pearson’s correlation coefficient for SUV, ADC, PDFF, R2*, and Spearman’s correlation coefficient for visual score.

### Correlation between top predictors and bone-marrow PET/MR features

**Figure 2:**
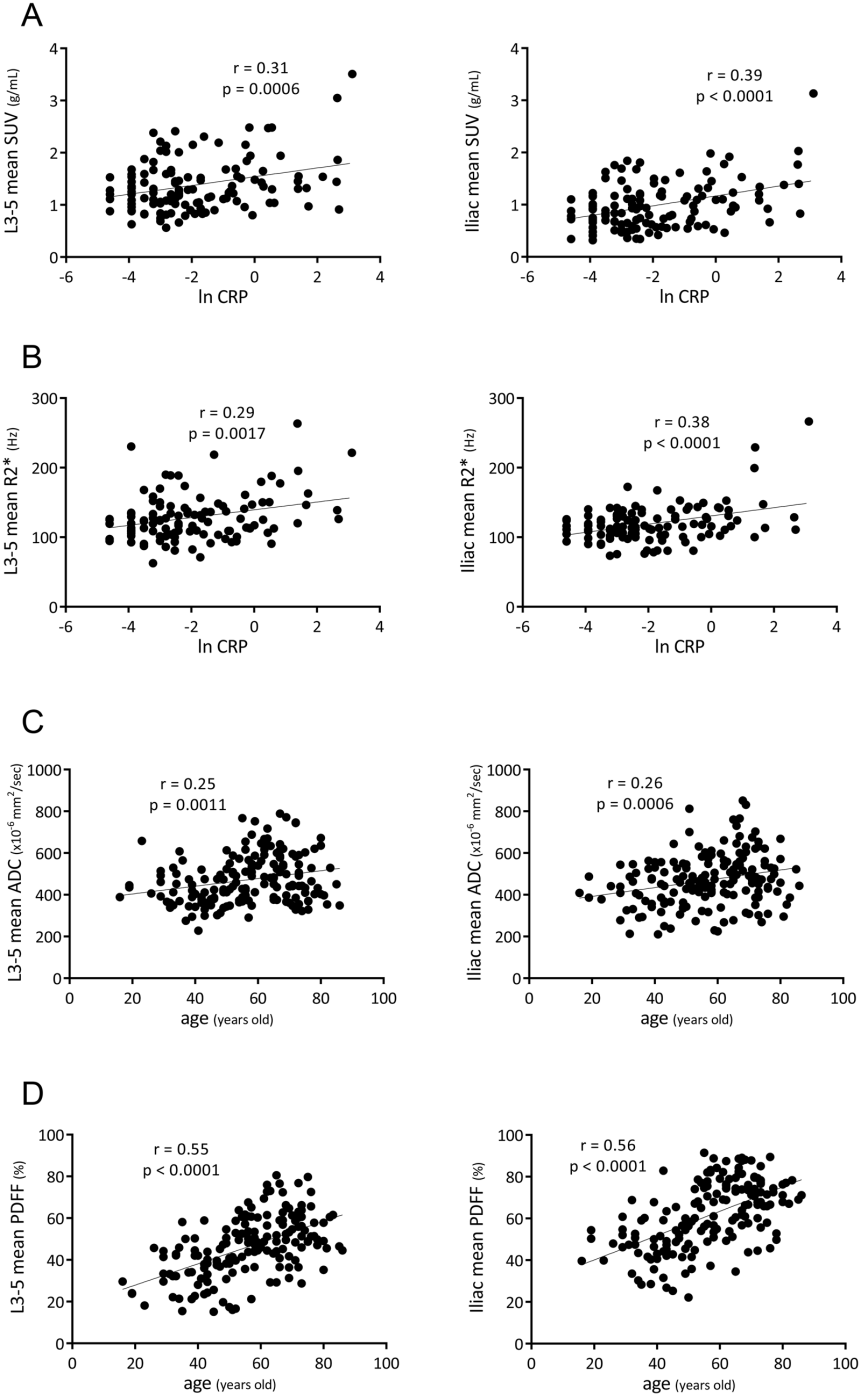
Correlation between top predictors and bone-marrow PET/MR features. Considering the non-normal distribution of CRP, a natural logarithm of CRP (ln CRP) was used. Regression lines of significance are shown with Pearson’s correlation coefficients (r) and associated p-values.

Bone-marrow [^18^F]FDG uptake and R2* were dependent on inflammation (CRP level) (Table 2). Considering the non-normal distribution of CRP, a natural logarithm of CRP (ln CRP) was correlated with bone-marrow SUV and R2* (Fig. 2A, B, respectively). Based on linear regression analyses, lumbar and iliac SUVs were positively correlated with ln CRP (Pearson’s r = 0.31, *p* = 0.0006, and r = 0.39, *p* < 0.0001, respectively), and lumbar and iliac R2* were positively correlated with ln CRP (r = 0.29, *p* = 0.0017, and r = 0.38, *p* < 0.0001, respectively). On the other hand, bone-marrow ADC and PDFF were strongly dependent on age (Table 2). Lumbar and iliac ADCs were positively correlated with age (r = 0.25, *p* = 0.0011, and r = 0.26, *p* = 0.0006, respectively) (Fig. 2C), and lumbar and iliac PDFFs were positively correlated with age (r = 0.55, *p* < 0.0001, and r = 0.56, *p* < 0.0001, respectively) (Fig. 2D).

### Correlation between bone-marrow SUV and MR features

**Figure 3:**
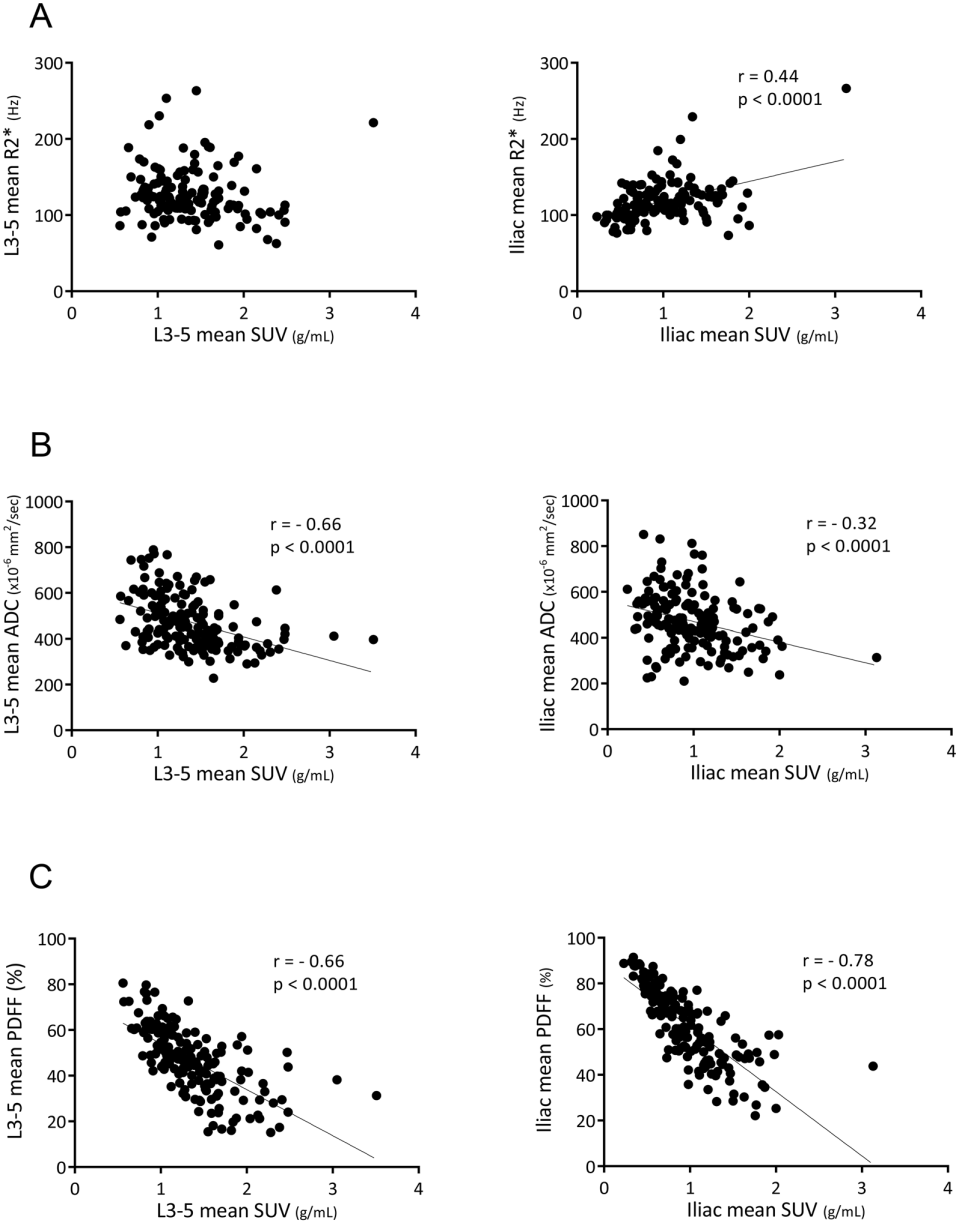
Correlation between bone-marrow SUV and MR features. Regression lines of significance are shown with Pearson’s correlation coefficients (r) and associated p-values.

Although lumbar SUV was not significantly correlated with lumbar R2*, iliac SUV was positively correlated with iliac R2* (r = 0.44, *p* < 0.0001) (Fig. 3A). In contrast, lumbar and iliac SUVs were negatively correlated with lumbar and iliac ADCs (r = − 0.66, *p* < 0.0001, and r = − 0.32, *p* < 0.0001, respectively) (Fig. 3B), and with lumbar and iliac PDFFs (r = − 0.66, *p* < 0.0001, and r = − 0.78, *p* < 0.0001, respectively) (Fig. 3C).

### Representative cases

**Figure 4:**
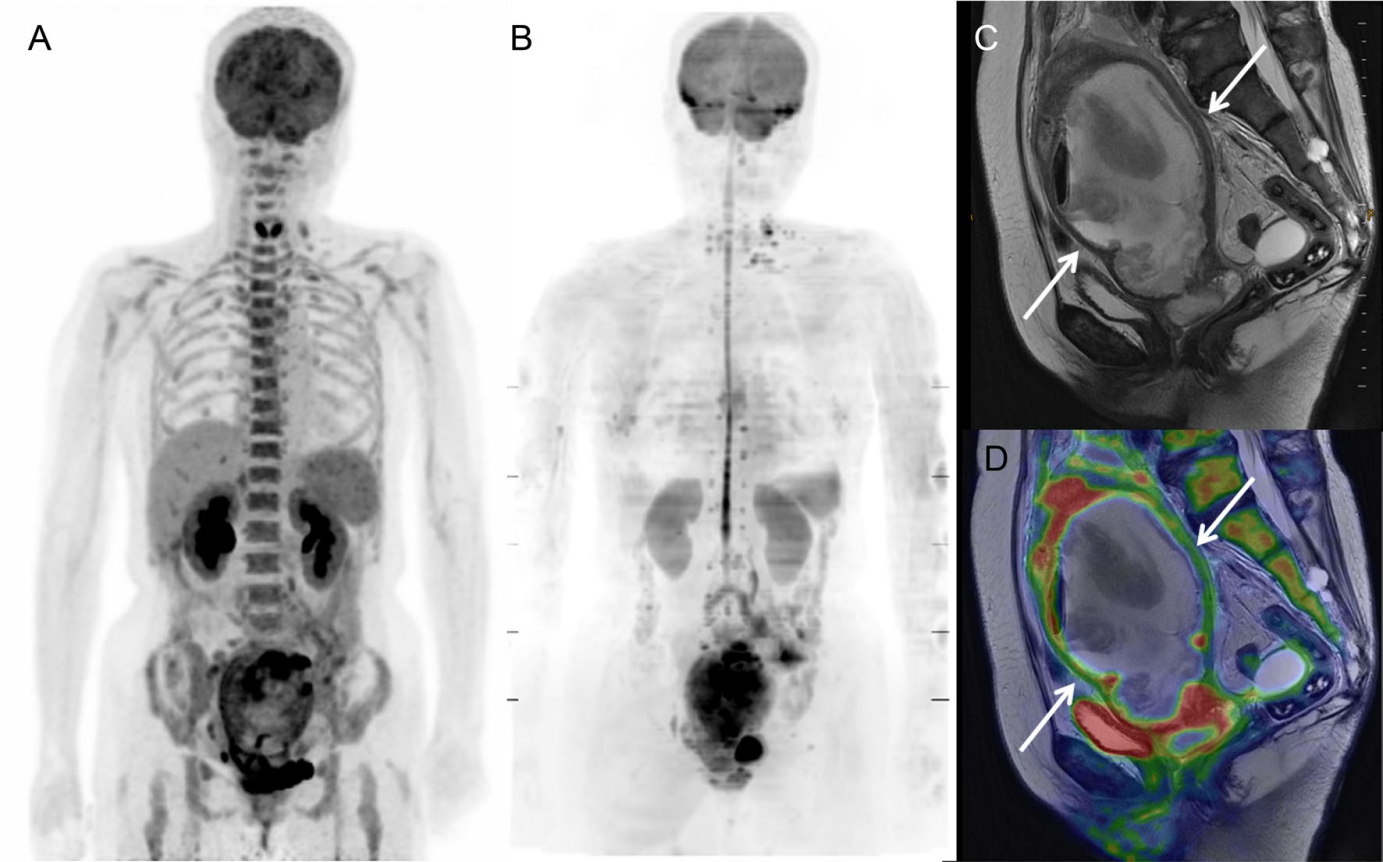
[^18^F]FDG PET and MR images of a 69-year-old woman with uterine cervical cancer and pyometra. Bone marrow exhibited high [^18^F]FDG uptake on PET (lumbar SUV = 3.51 g/mL) (**A**), whereas it was invisible on DWI with increased R2* (lumbar R2* = 221.3 Hz) (**B**). Due to obstruction by the primary tumor, severe pyometra was observed on T2-weighted and fusion images (**C** and **D**, respectively). Blood data revealed severe inflammation (Hb = 11.1 g/dL, WBC = 11.4 × 10^3^ /μL, and CRP = 22.5 mg/dL).

The [^18^F]FDG PET and MR images of a 69-year-old woman with uterine cervical cancer are shown in Fig. 4. Bone marrow demonstrated high [^18^F]FDG uptake on PET (lumbar SUV = 3.51 g/mL) (Fig. 4A), whereas it was invisible on DWI with increased R2* (lumbar R2* = 221.3 Hz) (Fig. 4B). Due to obstruction by the primary tumor, huge pyometra was observed on T2-weighted and fusion images (Fig. 4C, D, respectively). Blood data revealed severe inflammation (Hb = 11.1 g/dL, WBC = 11.4 × 10^3^/μL, and CRP = 22.5 mg/dL).

**Figure 5:**
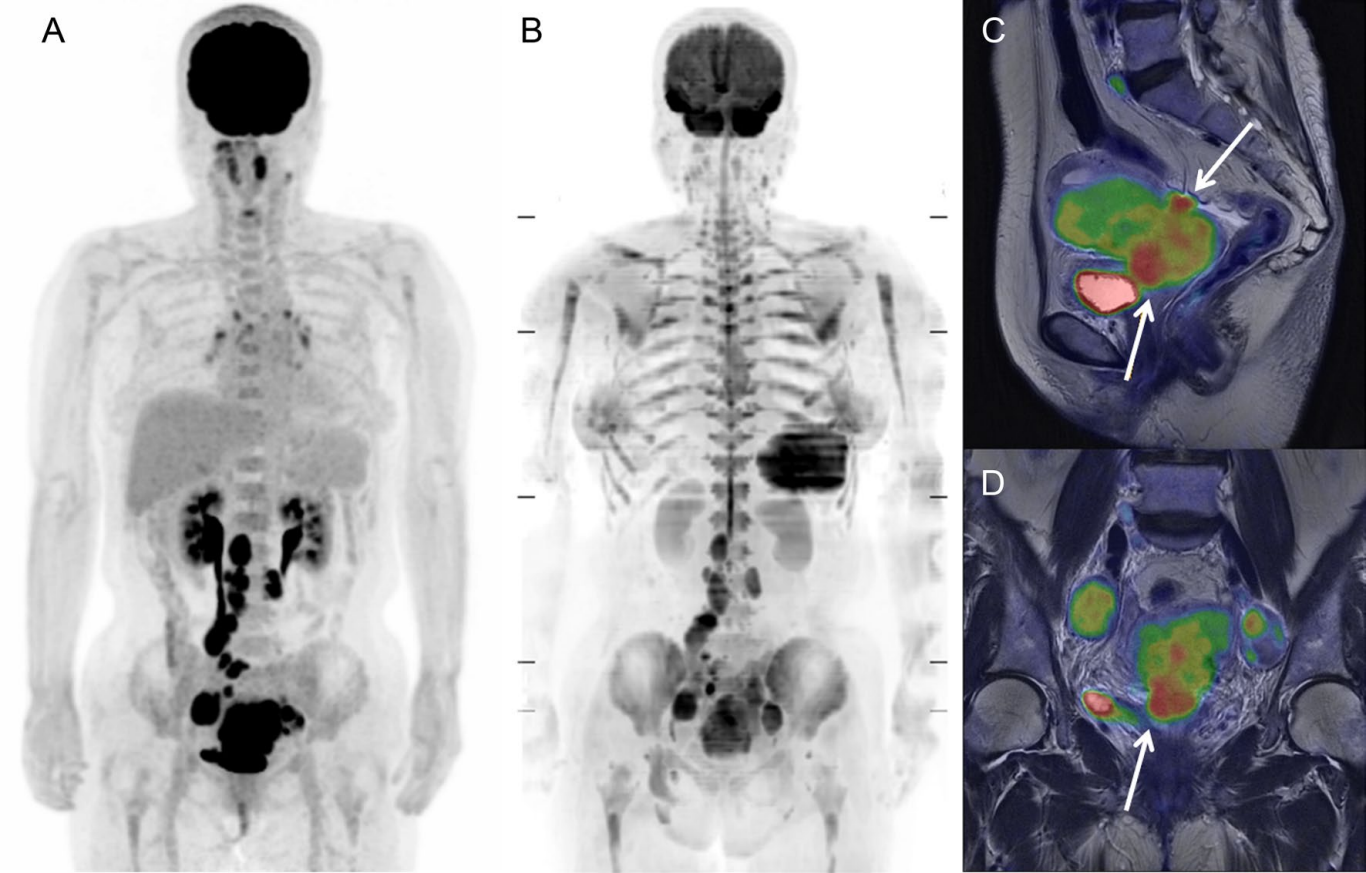
[^18^F]FDG PET and MR images of a 65-year-old woman with uterine cervical cancer and lymph-node metastases. Bone marrow exhibited normal [^18^F]FDG uptake on PET (lumbar SUV = 1.96 g/mL) (**A**) and high signal intensity on DWI (lumbar ADC = 376 mm^2^/sec) (**B**). Due to genital bleeding from the primary tumor (**C** and **D**), blood data revealed severe anemia and increased erythropoietic activity (Hb = 6.7 g/dL, RDW = 21.2%, WBC = 8.8 × 10^3^ /μL, and CRP = 0.08 mg/dL).

The [^18^F]FDG PET and MR images of a 65-year-old woman with uterine cervical cancer and lymph-node metastases are shown in Fig. 5. Bone marrow demonstrated normal [^18^F]FDG uptake on PET (lumbar SUV = 1.96 g/mL) (Fig. 5A) and high signal intensity on DWI (lumbar ADC = 376 mm^2^/sec) (Fig. 5B). Due to genital bleeding from the primary tumor, blood data revealed severe anemia and increased erythropoietic activity (Hb = 6.7 g/dL, RDW = 21.2%, WBC = 8.8 × 10^3^/μL, and CRP = 0.08 mg/dL).

## Discussion

To the best of our knowledge, this is the first study that has clearly identified and directly compared the factors influencing bone-marrow [^18^F]FDG uptake and MR signals using integrated PET/MRI. Bone-marrow [^18^F]FDG uptake and R2* were dependent on inflammation (CRP level), whereas bone-marrow ADC and PDFF were dependent on age, and DWI signals (visual scores) were dependent on erythropoietic activity (RDW).

Inoue et al. previously reported the association between bone-marrow [^18^F]FDG uptake and the CRP level^10^. Their multiple regression analyses revealed that the significant predictors of bone-marrow [^18^F]FDG uptake (mean SUV) were age and the CRP level in the benign group (n = 32), and the red blood cell (RBC) count and CRP level in the malignancy group (n = 33). Salaun et al. performed multivariate analyses and found independent correlations between bone-marrow [^18^F]FDG uptake visual grading and the CRP level (*p* = 0.007), and between sacral [^18^F]FDG SUVs and the CRP level (*p* = 0.032) in Hodgkin's lymphoma patients (n = 106)^11^. Our results regarding the relationship between bone-marrow [^18^F]FDG uptake and CRP level were consistent with the previous studies, and likely more precise because the present study included more patients (n = 177) and therefore provided more robust results from multiple regression analyses. Bone-marrow glucose metabolism is mainly regulated by granulocyte progenitors and stimulated by endogenous hematopoietic growth factors^9^.

**Figure 6:**
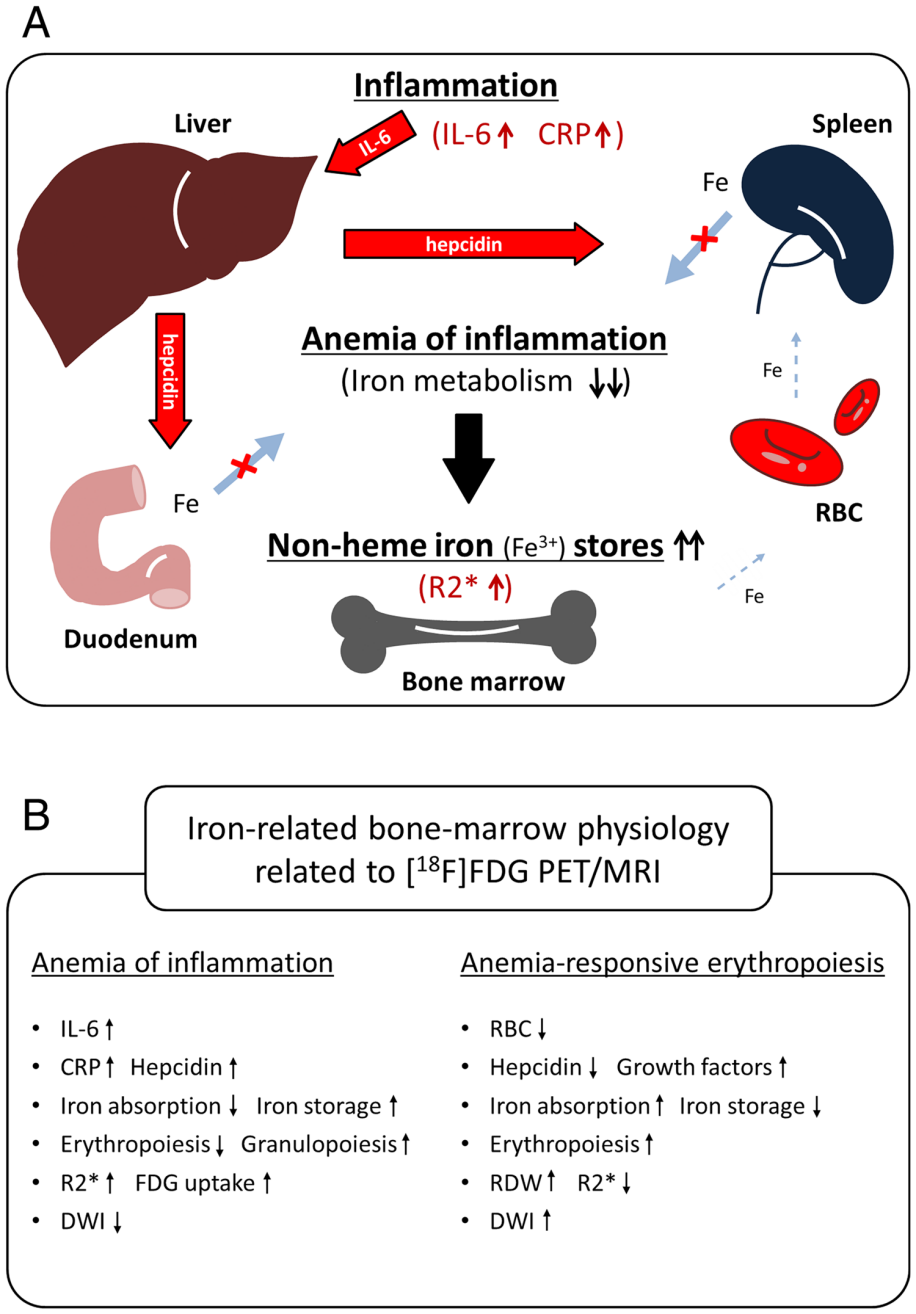
**A** Assumed relationship between bone-marrow R2* and CRP. Under inflammatory conditions, inflammatory cytokines, such as interleukin 6 (IL-6), induce excess synthesis of CRP and the iron-regulatory hormone hepcidin secreted by hepatocytes, which inhibits iron entry into plasma by binding to and inactivating the iron exporter ferroportin in target cells such as duodenal enterocytes and tissue macrophages. These conditions lead to inadequate erythrocyte production in the setting of low serum iron despite preserved or even increased macrophage iron stores in the bone marrow: anemia of inflammation. Stored iron exists in a state of non-heme Fe^3+^, such as ferritin and hemosiderin, which shortens the T2* relaxation time, resulting in an increased R2* relaxation rate. **B** Comparison of two distinct types of iron-related bone-marrow physiology related to [^18^F]FDG PET/MRI. In the setting of anemia of inflammation, inflammatory cytokines and hepcidin suppress iron absorption and erythropoiesis, and increase non-heme iron storage in the marrow, resulting in high R2* relaxation rates and decreased bone-marrow signals on DWI. Stimulated granulopoiesis in the marrow induces an increased [^18^F]FDG uptake. On the other hand, in a state of anemia-responsive erythropoiesis, iron metabolism and erythropoiesis (RDW) are increased, and iron storage (R2*) is decreased by hepcidin insufficiency, leading to an increase in bone-marrow DWI signals.

The direct correlation between bone-marrow R2* (iron deposition) and serum CRP level was demonstrated in this study. The assumed relationship between bone-marrow R2* and CRP is shown in Fig. 6A. Under inflammatory conditions, inflammatory cytokines, such as interleukin 6 (IL-6), induce excess synthesis of CRP and the iron-regulatory hormone hepcidin. Hepcidin is a small peptide hormone secreted by hepatocytes that inhibits iron entry into plasma by binding to and inactivating the iron exporter ferroportin in target cells such as duodenal enterocytes and tissue macrophages. These conditions lead to inadequate erythrocyte production in the setting of low serum iron despite preserved or even increased macrophage iron stores in the bone marrow. That is known as ‘anemia of inflammation’^17–19^. Stored iron exists in a state of non-heme Fe^3+^, such as ferritin and hemosiderin, which shortens the T2* relaxation time, resulting in an increase in the R2* relaxation rate and reduced DWI signals of bone marrow.

The recent report of Zeng et al. using [^18^F]FDG PET/MRI with DWI supports our hypothesis^6^. They presented a case with bone-marrow involvement of lymphoma which showed a great discrepancy between bone-marrow [^18^F]FDG PET and DWI. PET showed abnormally increased [^18^F]FDG uptake in nearly the entire bone marrow of the trunk and bilateral proximal extremities; in contrast, DWI showed significantly lower signal intensities in the entire bone marrow than those in the skeletal muscle probably due to iron deposition in bone marrow caused by posttransfusion iron overload. Careful attention is required to accurately evaluate bone-marrow disorders using [^18^F]FDG PET/MRI in distinction from physiological changes responsive to inflammation and iron deficiency.

Age was a predominant factor influencing bone-marrow PDFF and vertebral DWI signals, and a moderate factor influencing bone-marrow ADC (Table 2). Whole-body bone marrows contain hematopoietic cells at birth (red marrow); these are gradually replaced by fat tissue (yellow marrow), and the physiological conversion is completed by age 25. Red marrow is replaced by yellow marrow proximally to the axial skeleton. Lumbar and pelvic fat contents (PDFF) increased with age (Fig. 2D) and the result was consistent with the latest reports of Schmeel et al. and ours^4,7^. RDW was a predominant factor influencing bone-marrow DWI signals of pelvis and ribs (Table 2). RDW is a measure of the range of variations in RBC volumes and can offer an indirect measure of the erythropoietic activity of red bone marrow. As described in our recent report^7^, age, Hb, and RDW are the predominant predictors for bone-marrow signals on DWI. High bone-marrow signals on DWI are related to young age, anemia, and high RDW (anemia-responsive erythropoiesis).

A simplified comparison of two distinct types of iron-related bone-marrow physiology related to [^18^F]FDG PET/MRI is presented in Fig. 6B. In the setting of anemia of inflammation, inflammatory cytokines and hepcidin suppress iron absorption and erythropoiesis, and increase non-heme iron storage on the marrow resulting in high R2* relaxation rates and decreased bone-marrow signals on DWI. Stimulated granulopoiesis in the marrow induces an increased [^18^F]FDG uptake. On the other hand, in a state of anemia-responsive erythropoiesis, iron metabolism and erythropoiesis (RDW) are increased, and iron storage (R2*) is decreased by hepcidin insufficiency, leading to an increase in bone-marrow DWI signals.

In the present study, bone-marrow [^18^F]FDG uptake was significantly correlated with MR features (ADC, PDFF, and R2*) simultaneously obtained at the same coordinate positions by PET/MRI (Figs. 1, 3). In particular, bone-marrow [^18^F]FDG SUV showed significant negative correlations with bone-marrow ADC and PDFF at L3–5 and ilium, which is consistent partially with the report of Schraml et al.^20^. The inverse correlations of bone-marrow [^18^F]FDG uptake with ADC and PDFF may suggest that bone-marrow glucose metabolism is stimulated with increased bone-marrow cellular density and/or hematopoietic activity and that bone marrow adipose tissue does not have a comparable high metabolic activity as brown adipose tissue.

Our study had two limitations. First, although the predictive factors for bone-marrow [^18^F]FDG uptake and MR signals may be gender dependent^21^, the gender difference was not evaluated because the study population mainly consisted of women (with gynecological tumors). A larger number of male patients are required for the evaluation of the gender difference in future studies. Second, data collection allowed patients with CBC and CRP measured within one week of the scan. As these values can vary within days (especially CRP), blood data from the same day is preferable.

In conclusion, bone-marrow [^18^F]FDG uptake and R2* reflect anemia of inflammation (increased granulopoiesis and reduced iron metabolism), whereas bone-marrow DWI and PDFF reflect anemia-responsive erythropoiesis. Careful attention is required to accurately evaluate bone-marrow disorders using [^18^F]FDG PET/MRI in distinction from physiological changes responsive to inflammation and iron deficiency. Integrated [^18^F]FDG PET/MRI provides multi-parametric functional images, and demonstrates the iron-related bone-marrow physiology.

## Supplementary information

Supplementary file1

## Data Availability

Authors confirm that all relevant data are included in the article.

## Abbreviations

[^18^F]FDG: 2-[^18^F]-fluoro-2-deoxy-*D*-glucose
PDFF: Proton density fat fraction
R2*: Reciprocal of the T2* relaxation time
Hb: Hemoglobin
RDW: Red cell distribution width
WBC: White blood cell
CRP: C-reactive protein
GCSF: Granulocyte colony-stimulating factor
CBC: Complete blood count
eGFR: Estimated glomerular filtration rate
MR-AC: Magnetic resonance based attenuation correction
OSEM: Ordered subset expectation maximization
SUV: Standardized uptake value
EPI: Echoplanar imaging
STIR: Short inversion time inversion recovery
SSRF: Spectral-spatial radiofrequency
SNR: Signal-to-noise ratio
IDEAL-IQ: Iterative decomposition of water and fat with echo asymmetry and least-squares estimation quantitation sequence
ROI: Region of interest
MIP: Maximum intensity projection
Plt: Platelet count
PDW: Platelet distribution width
RBC: Red blood cell
IL-6: Interleukin 6
