# Supplementary material for: Integrated [18F]FDG PET/MRI demonstrates the iron-related bone-marrow physiology
Source: Sci Rep. 2020 Aug 17;10:13878. doi: 10.1038/s41598-020-70854-w (PMC7431424; doi:10.1038/s41598-020-70854-w)
Supplement: Supplementary file 1 — Supplementary file1 [file 41598_2020_70854_MOESM1_ESM.docx]

Title:

Integrated [^18^F]FDG PET/MRI demonstrates the iron-related bone-marrow physiology

Author list:

Tetsuya Tsujikawa^1)^, Hiroshi Oikawa^1)^, Toshiki Tasaki^2)^, Naoko Hosono^2)^, Hideaki Tsuyoshi^3)^, Mahmudur G. M. Rahman^1,5)^, Yoshio Yoshida^3)^, Takahiro Yamauchi^2)^, Hirohiko Kimura^4)^, Hidehiko Okazawa^1)^

Affiliation

^1)^ Biomedical Imaging Research Center, University of Fukui, Fukui, Japan

^2)^ Department of Hematology and Oncology, Faculty of Medical Sciences, University of Fukui, Fukui, Japan

^3)^ Department of Obstetrics and Gynecology, Faculty of Medical Sciences, University of Fukui, Fukui, Japan

^4)^ Department of Radiology, Faculty of Medical Sciences, University of Fukui, Fukui, Japan

^5)^ Department of Biomedical Engineering, Khulna University of Engineering and Technology, Khulna, Bangladesh

**Supplementary Table 1: MRI protocol for whole-body PET/MRI**

| **Parameters** | **Diffusion-weighted imaging** | **Proton density fat fraction R2*** | **T1-weighted imaging** | **T2-weighted imaging** |
| --- | --- | --- | --- | --- |
| Sequence type | Single-shot spin-echo EPI | IDEAL-IQ | LAVA-Flex | SSFSE |
| FOV (mm) | 576 x 345 | 500 x 300 | 500 x 400 | 500 x 300 |
| Matrix size | 128 x 128 | 256 x 192 | 300 x 200 | 384 x 256 |
| No. of slices | 40 | 34 | 120 | 40 |
| Slice thickness (mm) | 6 | 6 | 4 | 6 |
| Overlap (% of slice thickness) | 0 | 0 | 50 | 0 |
| TR (ms) | 5000 | 7.1 | 4.4 | 1600 |
| TE (ms) | 61 | 0.9-5.3 (6 echoes) | 1.3, 2.2 | 80 |
| Bandwidth (kHz) | 250 | 111.11 | 142.86 | 83.33 |
| Flip angle (°) | 90 | 3 | 12 | 90 |
| NEX | 4 | 0.75 | 1 | 1 |
| Fat suppression | STIR (+ SSRF in abdomen & pelvis) | NA | NA | NA |
| b Values (s/mm^2^) | 0, 800 | NA | NA | NA |
| Parallel acquisition | Asset 2 | Asset 2 | Asset 2 | Asset 2 |
| No. of beds | 5-6 | 5-6 | 5-6 | 5-6 |
| Acquisition time per bed (min:sec) | 2:30 | 0:20 | 0:13 | 1:03 |
